# Role of Uterine Artery Embolization in Pseudoaneurysm of Uterine Artery: A Rare Cause of Secondary Postpartum Hemorrhage

**DOI:** 10.7759/cureus.2220

**Published:** 2018-02-23

**Authors:** Saraswathy Subramaniam, Chandran Nadarajan, Mohd E Aziz

**Affiliations:** 1 Department of Radiology, School of Medical Sciences, Universiti Sains Malaysia, Health Campus, Kelantan, Malaysia

**Keywords:** uterine artery embolization, secondary postpartum hemorrhage, uterine artery pseudoaneurysm

## Abstract

Uterine artery pseudoaneurysm is an uncommon cause of secondary postpartum hemorrhage, although it carries a high mortality rate. The etiology includes vascular trauma during cesarean section, vaginal delivery, curettage or hysterotomy. We present a post-cesarean female who developed delayed hemorrhage and was diagnosed with left uterine artery pseudoaneurysm. Selective transcatheter arterial embolization was performed and the pseudoaneurysm was successfully obliterated. Angiographic embolization is a safe and efficient method of treatment of postpartum hemorrhage due to pseudoaneurysm in hemodynamically stable patients. Thus, it should be considered as a treatment option before opting for surgery in favorable cases.

## Introduction

Pseudoaneurysm is anextra-luminal collection of blood which is contained by the adventitia or surrounding perivascular soft tissue. It communicates with the flowing arterial blood through a defect in the arterial wall. It causes recurrent hemorrhage when it connects with the uterine cavity [1]. The absence of a three-layer arterial wall lining in the pseudoaneurysm differentiates it from a true aneurysm [2]. Pseudoaneurysm is prone to spontaneous rupture and may be fatal [3]. Risk of pseudoaneurysm rupture corresponds to the size and intramural pressure.

Diagnosis is based on both Doppler sonography and arteriography [4]. Transcatheter uterine artery embolization (UAE) is a highly effective technique for treating obstetric and gynecologic hemorrhage, including pseudoaneurysms [5]. We report a case of uterine artery pseudoaneurysm presenting as secondary postpartum hemorrhage one week, and again at three weeks, after cesarean section delivery that was managed successfully with embolization.

## Case presentation

A 26-year-old gravida 1 para 1 was transferred to our institution 32 days post-partum with symptoms of excessive bleeding per vagina. She had undergone an emergency c-section for prolonged second stage. Intraoperatively there was an extended tear at the left side, 3 cm downwards to the bladder base, which was secured. She was apparently asymptomatic for seven days post operation. She later developed excessive bleeding per vagina and was readmitted twice on Day 8 and Day 25 post-cesarean section and was managed by suction and curettage on both admission and tissues sent to pathology. Histopathological examination results, however, showed no evidence of retained pregnancy products. The patient presented again with excessive bleeding per vagina on Day 32 post-cesarean section. She had a healed Pfannenstiel scar, bulky and soft uterus, and the cervical os was closed. Her hemoglobin level was 7.4g/dl. She was stabilized with crystalloids, four units packed red blood cells, and started on broad-spectrum antibiotics.

**Figure 1:**
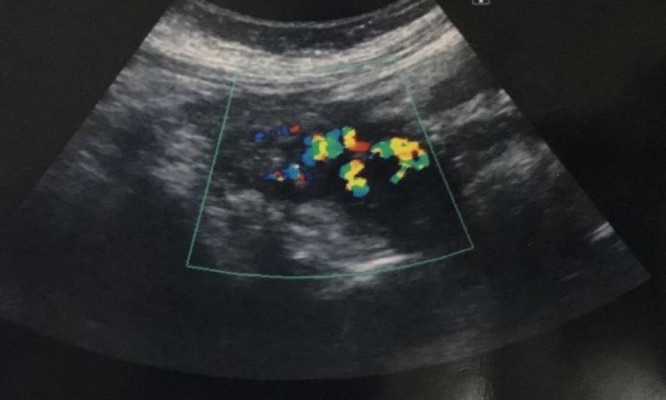
Color flow Doppler evaluation of the uterus demonstrating increased blood flow at left uterine artery region (blue box).

**Figure 2:**
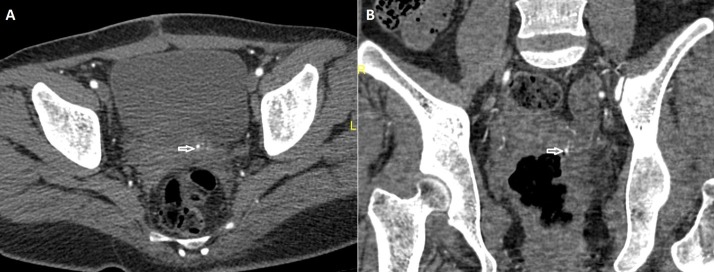
Coronal CT angiogram images of pelvis show contrast filling the pseudoaneurysm (arrowhead) within the uterus in relation to the left uterine artery.

Transabdominal ultrasonography revealed a postpartum uterus with blood clots within the uterine cavity. Hypoechoic lesion seen at the left wall of uterus and color flow Doppler sonography showed swirls of colors, which represents the opening of the pseudoaneurysm and its supplying artery (Figure 1). Pelvic computed tomography angiogram done with non-ionic contrast showed early contrast filling of the lesion within the uterus in relation to the left uterine artery (Figure 2A, 2B).

**Figure 3:**
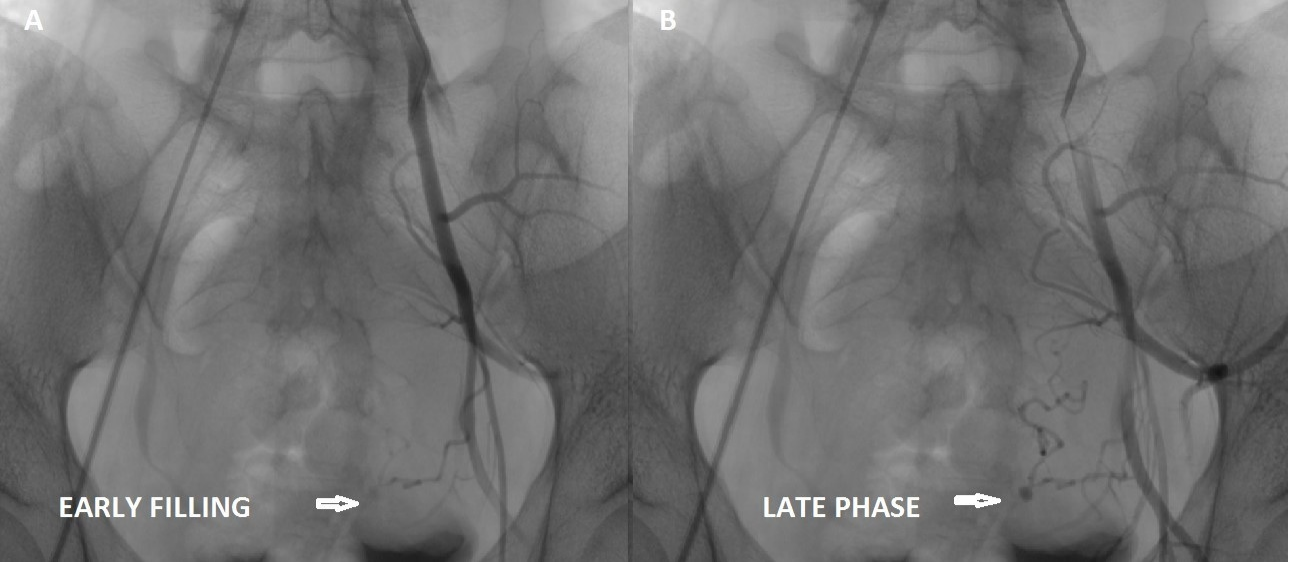
Selective left internal iliac angiogram: (A) Early and (B) late phase shows the pseudoaneurysm (arrows), arising from the tortuous left uterine artery.

**Figure 4:**
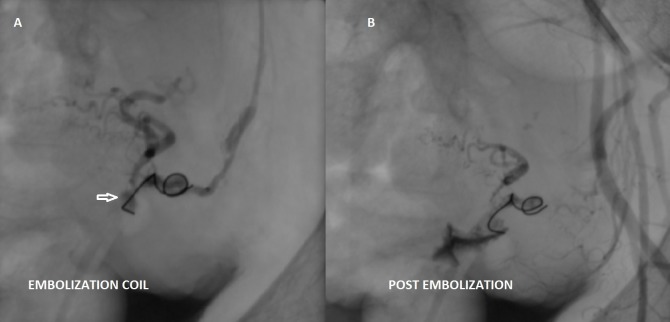
(A) Embolization coils (arrow) at the pseudoaneurysm. (B) Selective left internal iliac angiogram (post-embolization) using polyvinyl alcohol, glue and embolization coils show obliteration of the pseudoaneurysm.

**Figure 5:**
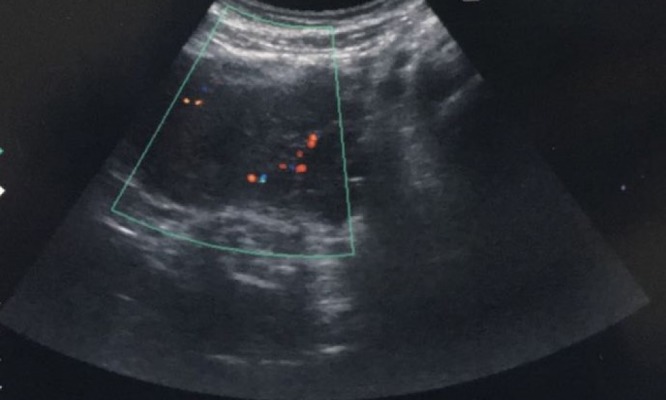
Follow up color Doppler ultrasound shows no evidence of blood flow within the pseudoaneurysm.

To preserve the fertility of this young patient, a transcatheter arterial embolization of this pseudoaneurysm was planned. She underwent digital subtraction angiography. Arteriography revealed a pseudoaneurysm from the terminal part of the left uterine artery, in addition, the left uterine artery was tortuous (Figure 3A, 3B). The left uterine artery was selectively embolized with a mixture of polyvinyl alcohol (PVA) and contrast media followed by one stainless-steel pushable coil 7 mm in diameter, and lastly, with a mixture of glue and lipiodol (Figure 4A). A post-embolization angiographic study was performed to ensure the complete occlusion of the vessels (Figure 4B). Follow-up color Doppler US showed no evidence of blood flow within the aneurysmal region (Figure 5).

## Discussion

Postpartum hemorrhage is a leading factor in maternal mortality and morbidity in developing countries. Secondary postpartum hemorrhage is excessive bleeding from 24 hours after delivery, up to six weeks postpartum. Causes include retained products of conception, endometritis, and placental bed subinvolution, pseudoaneurysm of uterine artery, arteriovenous malformations, and choriocarcinoma [6].

On ultrasound, pseudoaneurysms appear as an anechoic sac which shows turbulent arterial flow on Doppler. Doppler demonstrates to-and-fro sign in the neck of the pseudoaneurysm and yin-yang sign in the body of the pseudoaneurysm which is pathognomonic of pseudoaneurysm with a narrow neck. During systole, with higher arterial pressure, there is an influx of blood into the pseudoaneurysm. In diastole, the pressure in the artery drops and blood flows back through the pseudoaneurysm neck. This Doppler sign at the neck of the pseudoaneurysm together with turbulent flow helps to confirm the diagnosis [7].

Precise diagnosis of vascular causes of delayed postpartum hemorrhage helps to avoid unnecessary curettage for suspected retained products of conception and may avoid life-threatening blood loss. With the introduction of latest imaging modalities, the diagnosis of uterine artery pseudoaneurysm has become effortless [3]. Ultrasound and computed tomography imaging are being used routinely as initial diagnostic modalities. Angiography, however, remains the gold standard in diagnosing and treating vascular abnormalities.

In 1979, Brown, et al. reported the first successful case of selective arterial embolization for treatment in an extra-uterine pelvic hematoma after three failed surgical attempts. Since then, arterial embolization has been used widely to control postpartum bleeding with the success rate following embolization around 97% [8]. In a study of the efficacy of uterine artery embolization, Young Ho Choi reported 90% overall success rate with 10% complications [9]. In comparison, the reported success rates for internal iliac artery ligation vary from 42%–100% because of the extensive pelvic collateral circulation [10].

## Conclusions

For a woman with unexplained vaginal bleeding after C-section delivery, pseudoaneurysms are potentially life-threatening complications and should be considered in the differential diagnosis of secondary postpartum hemorrhage. Angiographic embolization should be considered as a treatment option before resorting to surgery in these cases.
